# Pupil dilation as an index of effort in cognitive control tasks: A review

**DOI:** 10.3758/s13423-018-1432-y

**Published:** 2018-02-12

**Authors:** Pauline van der Wel, Henk van Steenbergen

**Affiliations:** 1 Leiden University Institute of Psychology, Leiden, The Netherlands; 2 Leiden Institute for Brain and Cognition, Leiden, The Netherlands

**Keywords:** Pupil, Effort, Attention, Cognitive control, Inhibition, Switching, Updating, Behavioral performance, Resource allocation, Cognitive load

## Abstract

Pupillometry research has experienced an enormous revival in the last two decades. Here we briefly review the surge of recent studies on task-evoked pupil dilation in the context of cognitive control tasks with the primary aim being to evaluate the feasibility of using pupil dilation as an index of effort exertion, rather than task demand or difficulty. Our review shows that across the three cognitive control domains of updating, switching, and inhibition, increases in task demands typically leads to increases in pupil dilation. Studies show a diverging pattern with respect to the relationship between pupil dilation and performance and we show how an effort account of pupil dilation can provide an explanation of these findings. We also discuss future directions to further corroborate this account in the context of recent theories on cognitive control and effort and their potential neurobiological substrates.

## Introduction

“Face a mirror, look at your eyes and invent a mathematical problem, such as 81 times 17. Try to solve the problem and watch your pupil at the same time, a rather difficult exercise in divided attention. After a few attempts, almost everyone is able to observe the pupillary dilation that accompanies mental effort.” Daniel Kahneman, 1973, page 24.

Every day we encounter situations that demand goal-directed behavior and the control over our automatic, impulsive reactions. The ability to exert cognitive effort in these situations is highly important (Kahneman, 1973; Shenhav et al., 2017) and failures to do so can have consequences ranging from bad, e.g. failing an important math test as a student, to disastrous, e.g,. a traffic accident (Niezgoda, Tarnowski, Kruszewski, & Kamiński, 2015). Numerous recent studies have started to investigate the dilation of the human pupil under such conditions. This body of literature has started to provide important hints about the potential neurobiological mechanisms that underlie successful or failed recruitment of cognitive effort.

Although pupil dilation has been investigated since the early 1960s (Hess & Polt, 1964; Kahneman & Beatty, 1966), pupillometry research has experienced an enormous revival in the last two decades. In contrast to the early pioneering work, pupil dilation is relatively easy to study nowadays; eye trackers are relatively cheap and typically provide adequate temporal resolution and precision to detect even relatively small changes in pupil diameter. Although multiple reviews have already been published on the topic of pupil dilation and its link with a broad range of cognitive processes (Andreassi, 1980; Beatty & Lucero-Wagoner, 2000; Sirois & Brisson, 2014), such as attention (Laeng, Sirois, & Gredeback, 2012), memory (Goldinger & Papesh, 2012), and mental load (Just, Carpenter, & Miyake, 2003; Kramer, 1990), a comprehensive review on recent studies that have investigated pupil dilation in the context of cognitive control tasks is still missing. The current review aims to fill this gap. For a comprehensive review of older studies on the topic of pupil dilation and cognition, the reader is referred to earlier work (Beatty & Lucero-Wagoner, 2000; Just et al., 2003). The relationship between baseline pupil diameter and task performance (e.g., Tsukahara, Harrison, & Engle, 2016) is not covered in this review.

## Focus of the present review

The present review focusses on studies that have investigated pupil dilation during cognitive control tasks. In this context, pupil dilation refers to the stimulus-induced increase in pupil diameter relative to a pre-stimulus baseline period (also known as the task-evoked pupillary response; Goldinger & Papesh, 2012). Seminal studies have revealed that pupil dilation increases with increasing task demands. Two interpretations for this finding have been proposed: whereas some authors have simply concluded that pupil dilation thus reflects the demands or load by the task, others have taken it a step further and proposed that pupil dilation actually reflects the effort exerted in response to these demands (Hess & Polt, 1964; Kahneman, 1973; Kahneman & Beatty, 1966).

In light of recent renewed interest in cognitive effort in the fields of psychology and neuroscience (Kurzban, Duckworth, Kable, & Myers, 2013; Shenhav et al., 2017; Westbrook & Braver, 2015), a pressing aim for this review is to test the claim whether pupil dilation can be used as an independent index of effort. This is particularly important because, despite our increased understanding of the neural and computational mechanisms underlying cognitive effort, interpreting behavior in terms of cognitive effort risks circular reasoning (Navon, 1984; Shenhav et al., 2017). Take the case of the Stroop task. For this task, the difference in reaction time for incongruent versus congruent trials, i.e., the Stroop effect, is typically used as a measure of task performance. Without considering an independent measure of cognitive effort, it is hard to estimate from the mean reaction times how much effort the participant exerted. This is because a Stroop effect can simply be taken to reflect a successful indicator that task demands were increased and thus *required* cognitive effort, or alternatively, as an indication that *insufficient effort was recruited* to deal with the demand (Shenhav et al., 2017). Importantly, this issue pertains to all cognitive tasks that compare behavior in conditions that vary in task difficulty. Notably, the same reasoning can be applied to interpret effects of physiological signals in response to higher task demands. Thus, based on mean differences between easy and hard conditions alone it is impossible to decide whether physiological signals reflect mere task demand or effort exertion.

This review will take into account possible correlations between pupil dilation and task performance, because these might provide important cues to dissociate both accounts. Based on a definition of effort as the exertion of resources in the service of instrumental behavior (cf. Gendolla & Richter, 2010; Seery, 2013), if anything, effort should be associated with improved, not impaired task performance. However, we anticipate that not all studies show these effects because it is well-known that the exact relationship between effort and behavior depends on the specific context studied and that it can only be reliably investigated in a range where floor and ceiling effects in both measures are absent (Norman & Bobrow, 1975; cf. Hockey, 1997). In addition, correlations between pupil dilation and behavior can be investigated at different levels, that are sensitive to different confounds. For example, at the inter-individual level, participants with large pupil dilation might show better task performance than individuals with small pupil dilation, but it is important to demonstrate that such effects cannot be explained by differences in raw baseline pupil diameter caused by differences in ambient lighting (Beatty & Lucero-Wagoner, 2000), ethnicity (Quant & Woo, 1992), and age (MacLachlan & Howland, 2002), to name a few. This issue is particularly concerning in older studies that - instead of using subtraction – have expressed pupil dilation as the percent change from baseline pupil diameter, which might invalidate results (Beatty & Lucero-Wagoner, 2000). Alternatively, one might consider using pupil dilation difference scores based on within-subject comparisons, although these differences could reflect individual differences in the light reflex if stimulus luminance is not carefully controlled for. Therefore, evidence from intra-individual within-condition correlations that cannot be explained by confounding factors mentioned above will likely provide the most compelling evidence to test whether pupil dilation reflects effort.

This review is structured as follows: The following three sections first provide a brief literature review of studies that have investigated pupil dilation in relation to cognitive control. Sections are structured along the three domains of cognitive control identified by Miyake et al. (2000), discussing pupil dilation in relation to updating, switching, and inhibition respectively. These sections are followed by a critical evaluation of these findings and describe the underlying neural mechanisms that might underlie pupil dilation and effort. Finally, implications and directions for future research are discussed.

## Pupil studies on the updating component of cognitive control

The updating of working memory representations is an important aspect of cognitive control. It involves the monitoring of incoming information, the integration with information that is currently held in working memory and the updating in response to task demands (Miyake et al., 2000; Morris & Jones, 1990). Typical updating tasks are the letter memory task (Morris & Jones, 1990), the tone monitoring task (Binder et al., 1995), and the keep track task (Pylyshyn & Storm, 1988; Yntema, 1963). A few studies have combined these tasks with pupil measurements. For example, in the seminal study by Kahneman and Beatty (1967) using a tone monitoring task, it was shown that the pupil dilates more with increasing difficulty of pitch-discrimination. The authors therefore interpreted pupil dilation to reflect an increase in mental activity. More recent studies using the keep track task have shown increased pupil dilation with an increasing number of to be tracked objects (Alnæs et al., 2014; Wahn, Ferris, Hairston, & Ko, 2016; Wright, Boot, & Morgan, 2013).

In recent years, pupil dilation has been more commonly investigated in the context of mental arithmetic problems (e.g., Klingner, Tversky, & Hanrahan, 2011), the n-back task (Kirchner, 1958), the digit span task, and the Sternberg task (Sternberg, 1966), as reviewed in the following subsections.

### Mathematical problems

Mathematical problems have been used to investigate task-evoked pupil dilations and task difficulty by a bulk of studies. The seminal study on pupil dilation and mental arithmetic by Hess and Polt (1964) showed that the pupil dilates more with increasing difficulty, which was interpreted as that pupil dilation “can be used as a direct measure of mental activity.” Since then, this finding has been replicated by a large body of studies (Ahern & Beatty, 1979; Boersma, Wilton, Barham, & Muir, 1970; Bradshaw, 1968; Klingner et al., 2011; Payne, Parry, & Harasymiw, 1968; Schaefer, Ferguson, Klein, & Rawson, 1968). The majority of these studies have used multiplication problems, which evoke significantly more pupil dilation than adding problems (Jainta & Baccino, 2010). Boersma et al. (1970) reported that with increasing task difficulty overall pupil dilation increased, although at the later phase of the response period pupil dilation was actually larger for easy then for difficult stimuli, possibly reflecting diminished sustained engagement in the latter condition. Another study has revealed that relatively difficult auditory presented multiplication problems result in larger evoked dilation than relatively easy visual presentation of problems (Klingner et al., 2011).

Ahern and Beatty (1979) have revealed that the link between arithmetic performance and pupil dilation is moderated by individual differences in intelligence. Their study has shown that more intelligent individuals show smaller pupil dilation and respond more accurately for all levels of task difficulty as compared to less intelligent individuals. This finding suggests that intelligent individuals require less effort because they process information more efficiently. In contrast to these findings, a more recent study by van der Meer and colleagues (Van Der Meer et al., 2010) has reported that individuals with higher fluid intelligence solve an analogical reasoning task faster and more accurately, while also showing larger pupil dilations as compared to normal controls. The reason for the discrepancy between these two studies might be the different tasks involved: In contrast to the former study using an arithmetic task, the reasoning task was not highly overlearned thus making it more sensitive to differences in effort mobilization driven by variability in fluid intelligence. Consistent with this account, another study found that fluid intelligence was correlated with pupil dilation in an analogy task, whereas it was not significantly associated with pupil dilation in an algebra task (Bornemann et al., 2010).

### The n-back task

Another frequently used updating paradigm is the n-back task. The n-back task requires participants to indicate whether the currently presented letter or number is the same stimulus presented n trials back. Thus, task difficulty increases with increasing n (Kirchner, 1958). The n-back task is unique in its demands, because participants must constantly allocate attention to the task to perform well.

Multiple studies have shown that pupil dilation increases with increasing n (Belayachi et al., 2015; Brouwer, Hogervorst, Holewijn, & van Erp, 2014; Hopstaken, van der Linden, Bakker, & Kompier, 2015; Karatekin, Marcus, & Couperus, 2007; Niezgoda et al., 2015; Pehlivanoglu, Jain, Ariel, & Verhaeghen, 2014; Scharinger, Soutschek, Schubert, & Gerjets, 2015). Notably, a recent study using classification algorithms has shown that pupil dilation alone can be sufficient to distinguish high from low cognitive load up to an accuracy of 75 %. When compared to other measures such as electroencephalography, skin conductance, respiration, cardiac measures, and eye blinks, only the first exceeded this value with an classification accuracy of 80 % (Hogervorst, Brouwer, & van Erp, 2014). This finding shows that pupil dilation might be used as an index of cognitive load, thus is even suitable for applied contexts.

Regarding the link between pupil dilation and performance, one recent study has shown that an increased pupil dilation to the target stimuli of a 2-back task predicted improved performance in terms of a lower error rate (Rondeel, van Steenbergen, Holland, & van Knippenberg, 2015). This correlation at the inter-individual level suggests that pupil dilation reflects effort supporting improved performance.

### The digit span task

The digit span task consists of an encoding and a recall phase. In the encoding phase a string of digits of varying length is presented after which it must be recalled. Multiple studies have reported that the pupil dilates more with an increasing number of digits, showing that pupil dilation responds to increases in task demands (Granholm, Asarnow, Sarkin, & Dykes, 1996; Heitz, Schrock, Payne, & Engle, 2008; Johnson, 1971; Johnson, Singley, Peckham, Johnson, & Bunge, 2014; Kahneman & Beatty, 1966; Karatekin, 2004; Klingner et al., 2011; Peavler, 1974; Piquado, Isaacowitz, & Wingfield, 2010; Poock, 1973).

A handful of studies have investigated pupil dilation under conditions where task demands exceed the available cognitive resources. These studies have reported that when the amount of to be processed information exceeds one’s capacity, pupil dilation does no longer increase. For example, Poock (1973) showed that average pupil dilation drops below baseline under overload. More recent studies investigating the temporal dynamics of pupil dilation have revealed that pupil dilation reaches an asymptote up to a string of nine digits (Granholm et al., 1996) or 13 digits (Peavler, 1974), after which the pupil constricts. The exact timing of pupil dilation decline likely depends on whether instruction emphasizes maintaining active rehearsal under overload or not (Granholm et al., 1996). Two recent studies by Karatekin (2004) and Johnson, Singley, Peckham, Johnson, and Bunge (2014) have shown that children reach this asymptote at the level of six-digit strings.

### The Sternberg task

The Sternberg task (Sternberg, 1966) consists of an encoding and a search phase. In the encoding phase a string of digits of varying length is presented. After a delay of a few seconds, the search phase starts: Participants search in a new string for the letters that they have seen in the previous string. Task difficulty increases with increasing length of the digit string.

One pupillometry study on the Sternberg task has revealed that for young people two dilation peaks were present that increased with increasing task difficulty: one during the encoding and one during the search phase (Van Gerven, Paas, Van Merriënboer, & Schmidt, 2004). In older people, pupil dilation did not increase with increasing workload during the search phase. Notably, together with the absence of pupil dilation during the search phase, older participants also showed longer reaction times, whereas young participants did display increased pupil dilation and were faster. The authors interpreted the pupil as a sensitive measure of cognitive load, that might become less sensitive and thus less reliable with increasing age.

The influence of cognitive load has also been shown in a visual search task where pupil diameter increased for a more difficult version of the task and for larger search sets (Porter, Troscianko, & Gilchrist, 2007). More recent work has confirmed that pupil dilation increases with set size (Kursawe & Zimmer, 2015). Moreover, pupil size plateaus when set size exceeds capacity limitations (Unsworth & Robison, 2015, 2017b).

### Summary

To sum up, most studies using updating tasks have shown that pupil dilation increases with increasing task demands. When approaching working memory capacity limitations, pupil dilation plateaus or drops, depending on task instructions. Moreover, although not found in all studies (Ahern & Beatty, 1979), some studies on inter-individual differences have revealed that increased pupil dilation is associated with enhanced performance (Rondeel et al., 2015; Van Der Meer et al., 2010), suggesting that in these contexts pupil dilation might index the amount of effort allocated to the task at hand.

## Pupil studies on the shifting component of cognitive control

The ability to shift back and forth between tasks, operations, or mental sets is another important component of cognitive control. The plus-minus task (Jerslid, 1927; Spector & Biederman, 1976), the number-letter task (Rogers & Monsell, 1995), and the local-global task (Navon, 1977) have been identified as typical tasks that require shifting (Miyake et al., 2000). However, only the number-letter task has been related to pupil dilation. In this task participants respond to numbers according to changing tasks, which allows to compare task switch and task repetition trials. A study on pupil dilation using this switch task has revealed that pupil dilation increases during switch trials as compared to repetition trials (Rondeel et al., 2015), but this effect could not be related to individual differences in performance.

Another study has investigated whether pupil size changes when participants were forced to switch tasks or choose themselves to switch tasks (Katidioti, Borst, & Taatgen, 2014). This study has revealed that when a switch was forced, pupil size peaked briefly after the time of the switch. In contrast, self-initiated switches were preceded by pupil dilation a few seconds earlier. These findings suggest that self-initiated decision takes a relatively large amount of time and that this process might also involve preparatory effort.

In sum, there are few studies that have used switch tasks in combination with pupillometry. These studies show that the pupil is sensitive to changes in task difficulty and might also reflect effortful processes driving self-initiated switches. There is no clear evidence that links individual differences in pupil dilation to switching performance.

## Pupil studies on the inhibition component of cognitive control

Inhibition refers to the ability to overcome dominant or automatic responses in order to respond appropriately in a certain situation (Miyake et al., 2000). Miyake et al. (2000) has used the Stroop task (for an extensive review see Macleod, 1991; Stroop, 1935), the antisaccade task (Hallett, 1978), and the stop-signal task (Logan & Cowan, 1984) to measure inhibition ability. In addition to these tasks, other inhibition paradigms such as the flanker task (Eriksen & Eriksen, 1974), Simon task (Simon & Rudell, 1967), and Go/No-Go task have also been related to pupil dilation as reviewed below.

### The Go/No-Go task

Studies using the Go/No-Go paradigm have revealed that No-Go trials (requiring motor inhibition) evoke significantly smaller pupil dilations than Go trials (Reinhard & Lachnit, 2002; Richer, Silverman, & Beatty, 1983; Schacht, Dimigen, & Sommer, 2010; van der Molen, Boomsma, Jennings, & Nieuwboer, 1989). However, it is difficult to attribute pupil dilation effects of No-Go versus Go trials to differences in effort because they are confounded by the absence versus presence of motor execution, a process that may independently contribute to pupil dilation effects.

### The antisaccade task

Studies using the antisaccade task have shown that antisaccade trials requiring an eye movement to the opposite side of the presented target are associated with larger pupil dilations than prosaccade trials that require an eye movement to the target (Karatekin, Bingham, & White, 2010; Wang, Brien, & Munoz, 2015; Wang, McInnis, Brien, Pari, & Munoz, 2016). Additionally, larger pupil dilations were observed during the preparatory interval of antisaccades, as compared to the preparatory interval of correct prosaccades or erroneous prosaccades made in the anti-saccade condition. Notably, analyses on intra-individual correlations for the anti-saccade condition have revealed that larger preparatory dilations are associated with faster saccades (Wang et al., 2015, 2016).

### Conflict paradigms

Conflict tasks typically involve conflict between responses activated by the target and distractor stimuli, and cognitive control is needed to respond to the relevant information while inhibiting the irrelevant information (Botvinick et al., 2001). For example, in the Stroop task participants are asked to name the ink color whereas the word itself needs to be ignored, in the flanker task target information is surrounded by irrelevant distractors, and in the Simon task there is a match or mismatch between the spatial location of the target and the spatial location of the target key.

Many studies have shown that pupil dilation is increased for incongruent (conflict) trials as compared to congruent trials on the Stroop task (Brown et al., 1999; Brown, Steenbergen, Kedar, & Nieuwenhuis, 2014; Hasshim & Parris, 2015; Laeng, Ørbo, Holmlund, Miozzo, & Orbo, 2011; Rondeel et al., 2015; Siegle, Ichikawa, & Steinhauer, 2008; Siegle, Steinhauer, & Thase, 2004; Steinhauer, Siegle, Condray, & Pless, 2004) and the flanker task (Cohen, Moyal, & Henik, 2015; Geva et al., 2013; Scharinger, Soutschek, Schubert, & Gerjets, 2015; Van Bochove, Van der Haegen, Notebaert, & Verguts, 2013; Wendt, Kiesel, Geringswald, Purmann, & Fischer, 2014). Initial observations for the Simon task suggested an absence of conflict-induced pupil dilation (Schacht et al., 2010). However, more recent studies have shown increased pupil dilation on incompatible trials as compared to compatible trials also in this task (D’Ascenzo, Iani, Guidotti, Laeng, & Rubichi, 2016; Van Steenbergen & Band, 2013). Surprisingly, some studies have reported that the size of the Stroop effect (i.e., difference score of incongruent minus congruent conditions) in pupil dilation positively correlates with the Stroop effect in behavior, suggesting that increased pupil dilation correlates with impaired performance (Laeng et al., 2011; Rondeel et al., 2015). We will return to this finding in the next section of our review.

Some recent studies have also started to investigate pupil dilation in sequences of trials that allow to dissociate effects of current-trial conflict and previous-trial conflict (D’Ascenzo, Iani, Guidotti, Laeng, & Rubichi, 2016; Van Steenbergen & Band, 2013). The typical behavioral sequential effect observed in these paradigms is that the effect of conflict in the current trial is reduced (improved performance) if the previous trial was a conflict trial (Duthoo, Abrahamse, Braem, Boehler, & Notebaert, 2014; Egner, 2007; Gratton, Coles, & Donchin, 1992). This suggests that conflict triggers an adaptive increase in effort that can be measured in the subsequent trial. Conflict monitoring theory (Botvinick et al., 2001), posited that there are two dissociable processes and related neural systems that are involved in demand detection (conflict monitoring) and exertion of effort respectively. The original computational implementation of this model suggests that these processes are temporally dissociable, predicting different results for physiological signals that reflect task demands versus the allocation of effort.

Studies analyzing sequential analyses of pupil dilation have revealed smaller conflict-driven pupil dilations after incongruent than after congruent trials (D’Ascenzo, Iani, Guidotti, Laeng, & Rubichi, 2016; Van Steenbergen & Band, 2013). Following conflict monitor theory predictions, this suggest that pupil dilation reflects the demand for effort rather than the allocation of effort itself (van Steenbergen & Band, 2013). However, in contrast to the classic account (Botvinick et al., 2001), more recent work suggests that conflict monitoring and subsequent control adaptation are closely coupled in time (Shenhav, Botvinick, & Cohen, 2013). Therefore, sequential effects in pupil dilation could equally well be attributed to the allocation of effort (cf. Scherbaum, Fischer, Dshemuchadse, & Goschke, 2011). Notably, van Steenbergen and Band (2013) also observed intra-individual correlations suggesting that previous trial pupil dilation in conflict trials predicted speeded RT in the subsequent trial, which also seems consistent with an effort account of pupil dilation.

In a more recent study, measures of sequential adjustments in cognitive control were combined with a time-pressure manipulation in order to increase overall task demand (van Steenbergen, Band, & Hommel, 2015). Consistent with the assumed inverted U-shaped relationship between task difficulty and effort imposed by motivational limitations (Brehm & Self, 1989), overall increase in task demands abolished the effect of conflict on cognitive control adjustments in the subsequent trial. Moreover, under these high demands conditions, conflict produced an overall reduction in pupil dilation in the subsequent trial, possibly reflecting impaired exertion of effort in this task.

### Summary

In sum, many recent studies on inhibitory control have employed pupillometry. Studies using the antisaccade task and several conflict tasks have revealed a reliable increase in pupil dilation in conditions that require inhibitory control. Intra-individual correlations have indicated a link between pupil dilation and improved performance (van Steenbergen & Band, 2013; Wang et al., 2015, 2016). In contrast, associations investigated at the individual level have shown a positive correlation between the Stroop-effect in performance and pupil dilation (Laeng et al., 2011; Rondeel et al., 2015), suggesting that pupil dilation relates to impaired performance in these studies. We will address this inconsistency in turn.

## Evaluation of the reviewed literature

What do the studies reviewed above tell us about the feasibility to use pupil dilation as an index of cognitive effort? First, the literature reviewed above clearly shows that increases in task demands generally led to increases in pupil dilation across the cognitive control domains of updating, switching, and inhibition. However, as discussed at the outset of this review, based on these findings alone it cannot be decided whether this reflects simply an increase in task demands or an increase in actual effort exertion.

Given these considerations, one of the aims of this review was to document available correlations between pupil dilation and task performance. Although most studies did not report correlations and null-effects are hardly informative as explained earlier (Norman & Bobrow, 1975), it is worthwhile to elaborate on the studies that actually did report significant correlations. These correlations were observed at two levels. First, a limited number of studies have investigated correlations at the intra-individual level (van Steenbergen & Band, 2013; Wang et al., 2015, 2016). These studies consistently suggest that pupil dilation is associated with improved behavior in conditions that require inhibitory control. As alluded to earlier, these findings are particularly compelling because analyses at the intra-induvial levels are the least sensitive to confounds. Second, studies on intra-individual correlations are more diverging. Consistent with an effort account of pupil dilation, some of the studies suggested that individuals with increased pupil dilation actually showed better task performance than individuals with smaller pupil dilation (Rondeel et al., 2015; Van Der Meer et al., 2010). These findings support an account favoring pupil dilation as an index of effort exertion whereas these findings are hard to reconcile with the idea that pupil dilation simply reflects increased demands or task load only. On the other hand, some of the reviewed studies actually show a negative correlation between pupil dilation and performance. For example, two studies have shown a correlation between the Stroop effect in RTs and pupil dilation (Laeng et al., 2011; Rondeel et al., 2015). At face value, these observations better fit a task demand interpretation of pupil dilation. By this account, bad Stroop performers show physiological signatures of task demand due to alleged control impairments in these individuals. It is important to notice however that these findings can equally well be explained by an effort account. Accordingly, bad performers show increased pupil dilation because they try harder in order to compensate for their impaired inhibitory control – although this is unsuccessful (cf. Hockey, 1997). Alternatively, findings could be taken to reflect that good performers process information more efficiently and therefore exert less effort (cf. Ahern & Beatty, 1979). Taken together, we think that these preliminary findings show that the effort account could have more explanatory power and might therefore be preferred over an account that pupil dilation reflects mere task demands.

However, it goes without saying that it is hard to draw strong conclusions concerning the interpretation of pupil dilation, since it at best reflects a psychophysical marker that can only be reliable interpreted in restricted contexts (see the instructive caveats about the use of psychophysiological measures by Richter & Slade, 2017). Well-controlled lab conditions are always required to prevent alternative explanations – including for example matched luminance levels for the different demands in order to rule out an effect on the pupillary light reflex (Beatty & Lucero-Wagoner, 2000) and matched numbers of trials to exclude interpretations in terms of an orienting response or surprise (Braem, Coenen, Bombeke, van Bochove, & Notebaert, 2015). In addition, future studies should also start to carefully control for confounding factors such as age and ethnicity when relating pupil dilation and performance at the inter-individual level. To further advance our account, one might run crucial experiments that contrast predictions from both accounts in a single study. Such a study might include manipulations of task demands that range from easy to extremely difficult (cf. Richter, Friedrich, & Gendolla, 2008). Although demand and effort typically co-vary when success is possible and worthwhile, extremely high task demands are expected to produce disengagement and withdrawal of effort (Brehm & Self, 1989). Testing whether pupil dilation under these conditions of high task difficulty decreases or increases may provide important evidence for the effort or task load account respectively.

An alternative approach to further corroborate an effort account of pupil dilation would be to show that pupil dilation effects converge with other measures related to effort. For example, in another line of research, myocardial sympathetic activity has been proposed as the operational definition of effort mobilization, as primarily reflected in measures of systolic blood pressure and the pre-ejection period (Wright, 1996). Indeed numerous studies have shown that these measures are sensitive to task difficulty manipulations consistent with a role of effort mobilization (Gendolla, Wright, & Richter, 2011; Richter et al., 2008). We recently developed a method to measure a proxy of the pre-ejection period at a single-trial level. This method makes it possible to analyze cardiac effort as a task-evoked response (Kuipers et al., 2017), and to run analyses similar to those used in pupillometry studies (Spruit, Wilderjans, & van Steenbergen, 2018). Future studies are needed to determine whether pupil dilation data indeed converges with these cardiovascular measures of effort.

The activity of facial muscles has also been proposed to reflect effort (e.g., Boxtel & Jessurun, 1993; Cacioppo, Petty, & Morris, 1985; de Morree & Marcora, 2010). In particular, the facial corrugator muscle has been shown to respond to increases in cognitive demands. Notably, responses in the corrugator muscle have also been used as a measure of negative affect (e.g., Larsen, Norris, & Cacioppo, 2003) and thus might reflect the anxiety associated with task difficulty (uncertainty) and/or the intrinsic cost of cognitive control and effort exertion (Botvinick, 2007; Dreisbach & Fischer, 2016; Inzlicht, Bartholow, & Hirsh, 2015; van Steenbergen, 2015). Although the effect of response conflict on the corrugator seems not easy to identify (Schacht et al., 2010), it is sensitive to error commissions (Elkins-Brown, Saunders, He, & Inzlicht, 2017; Elkins-Brown, Saunders, & Inzlicht, 2016; Lindström, Mattsson-Mårn, Golkar, & Olsson, 2013) and it has been related to subsequent performance adjustments (Lindström et al., 2013). Similar findings have been observed for the pupil dilation response to errors (Murphy, Van Moort, & Nieuwenhuis, 2016). Importantly, pupil dilation to stimuli with negative valence has also been observed in other studies (e.g., Chiesa, Liuzza, Acciarino, & Aglioti, 2015), although it likely reflects the arousal and not the valence dimension of affective states (Bradley, Miccoli, Escrig, & Lang, 2008; Snowden et al., 2016; Van Steenbergen, Band, & Hommel, 2011). Thus, it remains an important topic for future studies to compare the convergence between the pupil dilation response and facial EMG in the context of cognitive control tasks.

## Neurobiological mechanisms

There is an increasing body of evidence that has started to reveal the brain mechanisms that underlie effort-related pupil dilation. The size of the pupil is controlled by two muscles, the sphincter and dilator muscles, which are differentially influenced by activity in the parasympathetic and sympathetic branches of the nervous system, respectively. Sympathetic activity drives the dilator muscle evoking dilation, whereas inhibition of parasympathetic activity reduces constriction of the sphincter muscle, which also results in dilation (Beatty & Lucero-Wagoner, 2000). These systems in turn reflect activity of neuromodulatory brain systems involving catecholamines and acetylcholine (Aston-Jones & Cohen, 2005; Verguts, Vassena, & Silvetti, 2015).

The modulations in arousal that drive pupil dilation in response to task demands are related to the locus-coeruleus norepinephrine (LC-NE) system. More specifically, it has been proposed that the LC receives input from brain areas that detect task demands such as the ACC, and that its efferent projections influences the levels of neural gain throughout the cortex, including frontal and parietal regions important for cognitive control (Aston-Jones & Cohen, 2005; Nieuwenhuis, De Geus, & Aston-Jones, 2011) and subcortical areas, such as the superior colliculus, important for attention (Foote & Morrison, 1987). Importantly, LC activity closely correlates with pupil diameter likely via common input from the gigantocellularis nucleus of the ventral medulla (Joshi, Li, Kalwani, & Gold, 2016). A growing number of studies have therefore used pupil diameter as an indirect index of the activity of the LC-NE system. Neurons in the LC-NE system have tonic and phasic modes of firing and task-evoked pupil dilation to a stimulus is driven by the latter. Notably, the phasic response of LC-NE neurons depends on its tonic mode and is strongest at intermediate levels of tonic firing. This mechanism suggests an inverted-U shape relationship between tonic LC activity and task-evoked arousal, which might also underlie individual differences in working memory capacity and effort (Unsworth & Robison, 2017a).

Similarly, acetylcholine has been proposed to play an important role in compensatory effort to keep up task performance under challenging conditions (Sarter, Gehring, & Kozak, 2006). Acetylcholine neurons originating from the basal forebrain have been shown to upregulate prefrontal areas in response to cognitive demands and these neurons also receive projections from frontal and midbrain areas, thus being in an ideal position to control motivated behavior (Sarter et al., 2006). Interestingly, a recent study has shown that activity in both norepinephrine and acetylcholine axons are reflected by pupil dilation, although they differ in temporal dynamics (Reimer et al., 2016).

The norepinephrine and acetylcholine systems receive projections from the anterior cingulate cortex which has been proposed to monitor the environment for cognitive conflict and demands (Botvinick et al., 2001), and to optimize the amount of effort based on its associated costs and expected payoffs coded in other regions of the brain such as the insula, ventral prefrontal cortex, striatum, and midbrain (Shenhav et al., 2013). These neural circuits thus support the amount of effort or control invested in a task, and the underlying processes are likely better captured by a decision process rather than by a capacity or resource that is physically limited (Kurzban et al., 2013; Shenhav et al., 2017). From this perspective, cognitive control shares many processes that are also important for value-based decision making (Berkman, Hutcherson, Livingston, Kahn, & Inzlicht, 2017), and it remains an important aim for future studies to use physiological measures including pupil dilation to better characterize the effort processes determined by these brain systems.

## Conclusion

The studies reviewed here show that pupil dilation can be used as an indirect index of effort in cognitive control tasks. Across the domains of updating, switching and inhibition, studies show that pupil dilation closely responds to changes in task demands and in some cases predict improved task performance. Future work that integrates other physiological indices of cognitive effort with neuroimaging techniques is needed to advance our understanding of the intricate role of brain and body mechanisms that underlie cognitive effort.
